# Supplementary material for: Using spatial statistics to infer game-theoretic interactions in an agent-based model of cancer cells
Source: bioRxiv. 2025 Jul 15:2025.07.09.664005. Preprint. [Version 1] doi: 10.1101/2025.07.09.664005 (PMC12338505; doi:10.1101/2025.07.09.664005)
Supplement: Supplement 1 [file media-1.pdf]

Using spatial statistics to infer game-theoretic interactions in an agent-based model of cancer cells

Sydney Leither[1,2◕*], Maximilian A. R. Strobl[3◕] Jacob G. Scott[3,4,5], Emily Dolson[1,2],

1 Department of Computer Science and Engineering, Michigan State University, East Lansing, Michigan, United States
2 Program in Ecology, Evolution, and Behavior, Michigan State University, East Lansing, Michigan, United States
3 Translational Hematology & Oncology Research, Cleveland Clinic, Cleveland, Ohio, United States
4 Department of Physics, Case Western Reserve University, Cleveland, Ohio, United States
5 Case Western Reserve University School of Medicine, Cleveland, Ohio, United States

◕These authors contributed equally to this work.
* leithers@msu.edu

1 Spatial statistics

1.1 Neighborhood composition

The neighborhood composition (NC) spatial statistic quantifies the average neighborhood structure around each cell of a given type. NC is calculated as follows: for each cell of type A, record the fraction of type B cells in their local neighborhood. Remove any data points where the fraction of type B is zero. The resulting data set is the distribution of the fraction of cell type B in the neighborhood of each cell type A. In this work, the local neighborhood size for calculating NC was set to 3.

Spatial Statistic	Type	Description	Source
Average Nearest Neighbor Index (ANNI)	Value	Clustering of cell types based on expected nearest neighbor distances	[1]
Cross Pair Correlation (CPCF)	Function	Aggregation of cell types across annuli	[2]
Cross Ripley's k	Function	Co-localization of cell types across radii	[3]
Entropy	Value	Shannon entropy of cell type A	[4]
KL Divergence	Value	Kullback-Leibler divergence between kernel density estimations of cell types	[5], [6]
Global Moran's i	Value	Spatial autocorrelation of continuous "proportion of cell type A in hex" over the whole population	[7]
Local Moran's i	Distribution	Spatial autocorrelation of continuous "proportion of cell type A in hex" in local neighborhoods	[7]
Nearest Neighbor (NN)	Distribution	Distances from each cell type A to any cell type B	[8]
Neighborhood Composition (NC)	Distribution	Fraction of cell type A in each cell type B's neighborhood	This paper
Proportion Sensitive	Value	Proportion of sensitive cells	This paper
Standard Effect Size of Quadrant Correlation Matrix (SES)	Value	Correlation between counts of cell types across regions	[9]
Wasserstein	Value	Wasserstein distance between 1D-projected spatial data of two cell types	[10]

Table 1. Spatial statistics used in this work. Implementations of each spatial statistic not sourced from this paper are from MuSpAn [11]. Parameters of each spatial statistic were set to MuSpAn's default values divided by 10, as MuSpAn expects images around size 1000x1000 and our images were of size 100x100.

2 Correlated feature clusters

**Chosen Feature**	**Features in Correlated Cluster**
ANNI RS	
ANNI SR	
CPCF RR Min	
CPCF RR Max	
CPCF SR Min	CPCF RS Min, Ripleys k RS Min, Ripleys k SR Min
CPCF RS Max	
CPCF SR Max	
CPCF SS Min	
CPCF SS Max	
Entropy	
Global i Resistant	
Global i Sensitive	
KL Divergence	
Local i Resistant Mean	
Local i Resistant SD	
Local i Resistant Skew	
Local i Sensitive Mean	
Local i Sensitive SD	
Local i Sensitive Skew	
Proportion Sensitive	NC RS Mean, NC RS Skew, NC SR Mean, NC SR Skew, NN RS Mean, NN RS SD, NN RS Skew, NN SR Mean, NN SR SD, NN SR Skew, , Ripleys k RR Min, Ripleys k RR Max, Ripleys k SS Min, Ripleys k SS Max
NC RS SD	
NC SR SD	
Ripleys k RS Max	
Ripleys k SR Max	
SES	
Wasserstein	

Table 2. Correlated feature clusters. Features are considered correlated if their Spearman's rank correlation coefficient $\rho > 0.9$. From each correlated cluster, we only analyze the chosen feature in this work.

3 Pairwise game distributions

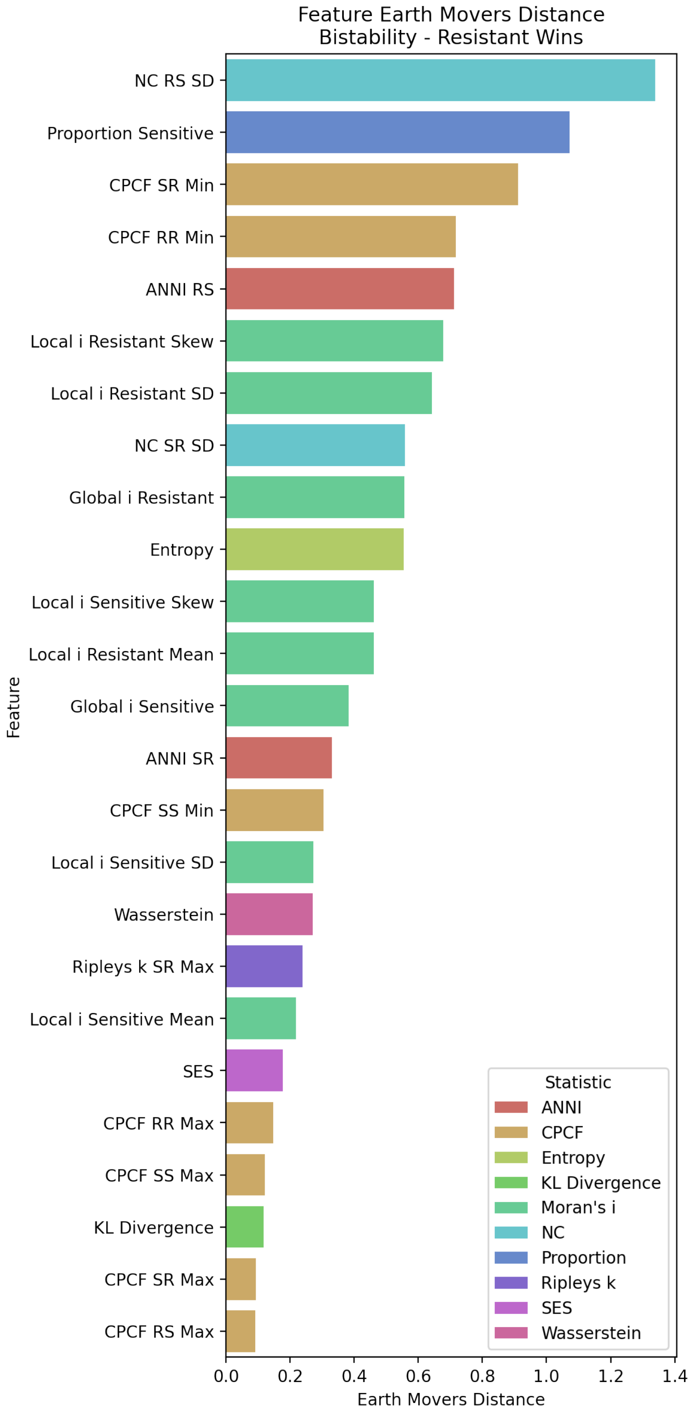

Fig 1. Full list of the distinguishability (Earth movers distance) between the feature distributions of *Bistability* and *Resistant Wins*.

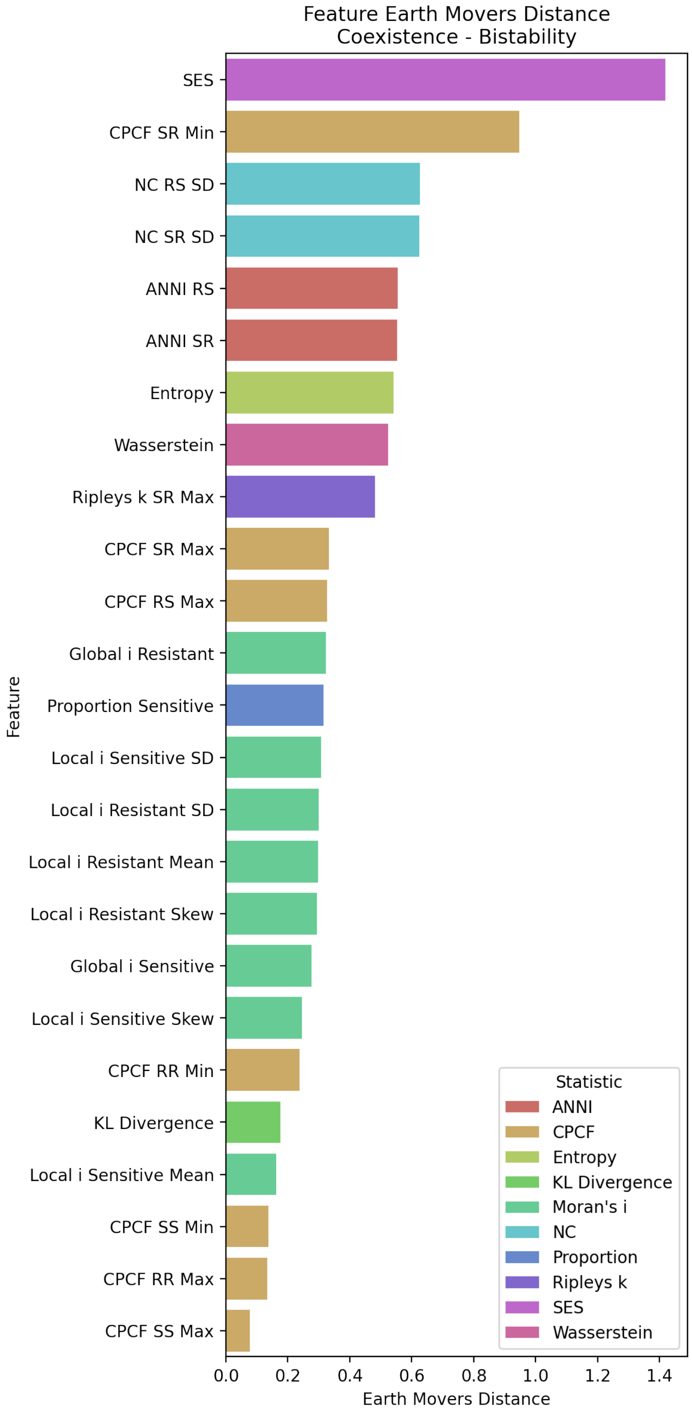

Fig 2. Full list of the distinguishability (Earth movers distance) between the feature distributions of *Coexistence* and *Bistability*.

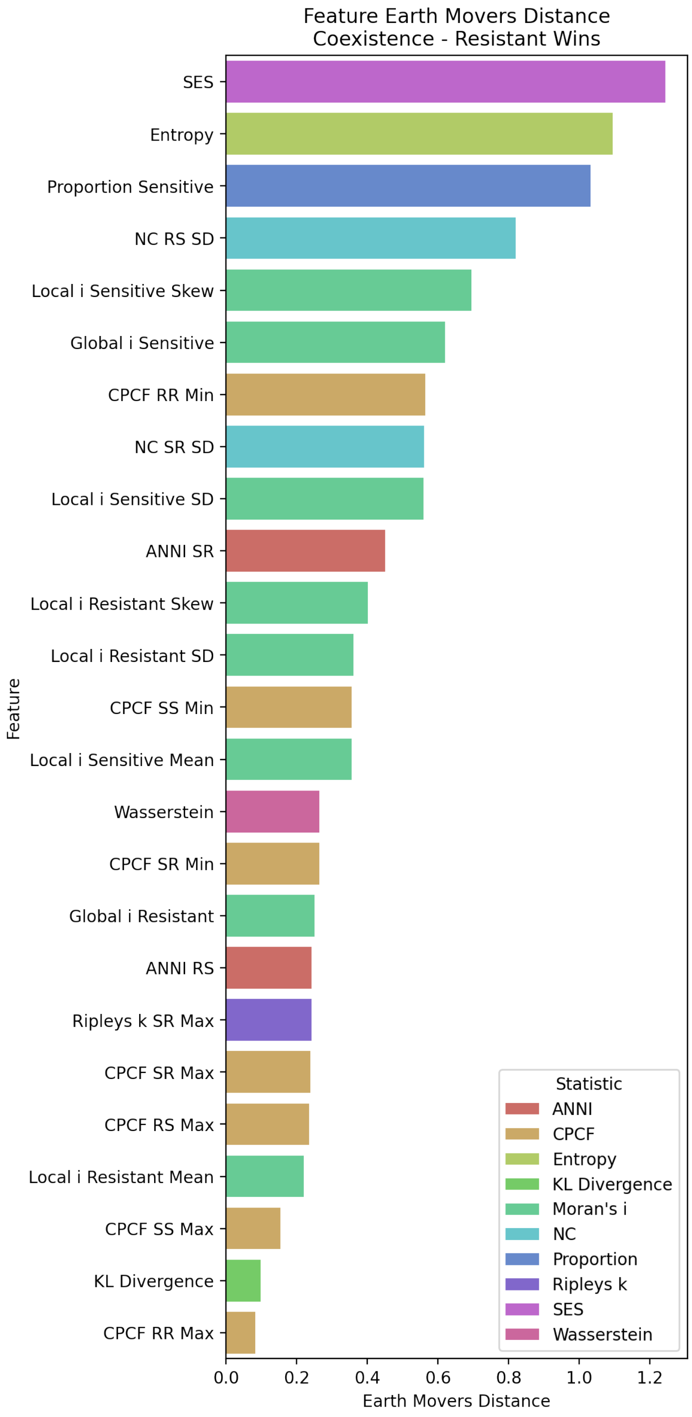

Fig 3. Full list of the distinguishability (Earth movers distance) between the feature distributions of *Coexistence* and *Resistant Wins*.

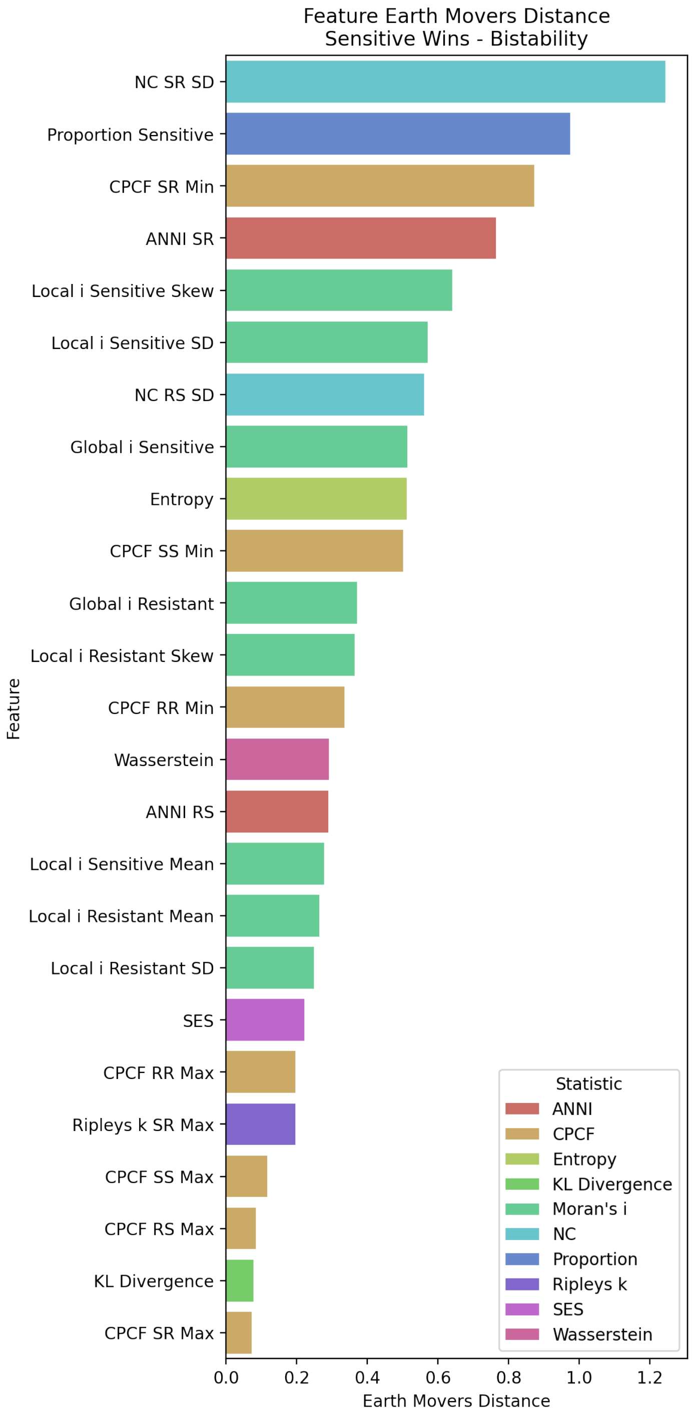

Fig 4. Full list of the distinguishability (Earth movers distance) between the feature distributions of *Sensitive Wins* and *Bistability*.

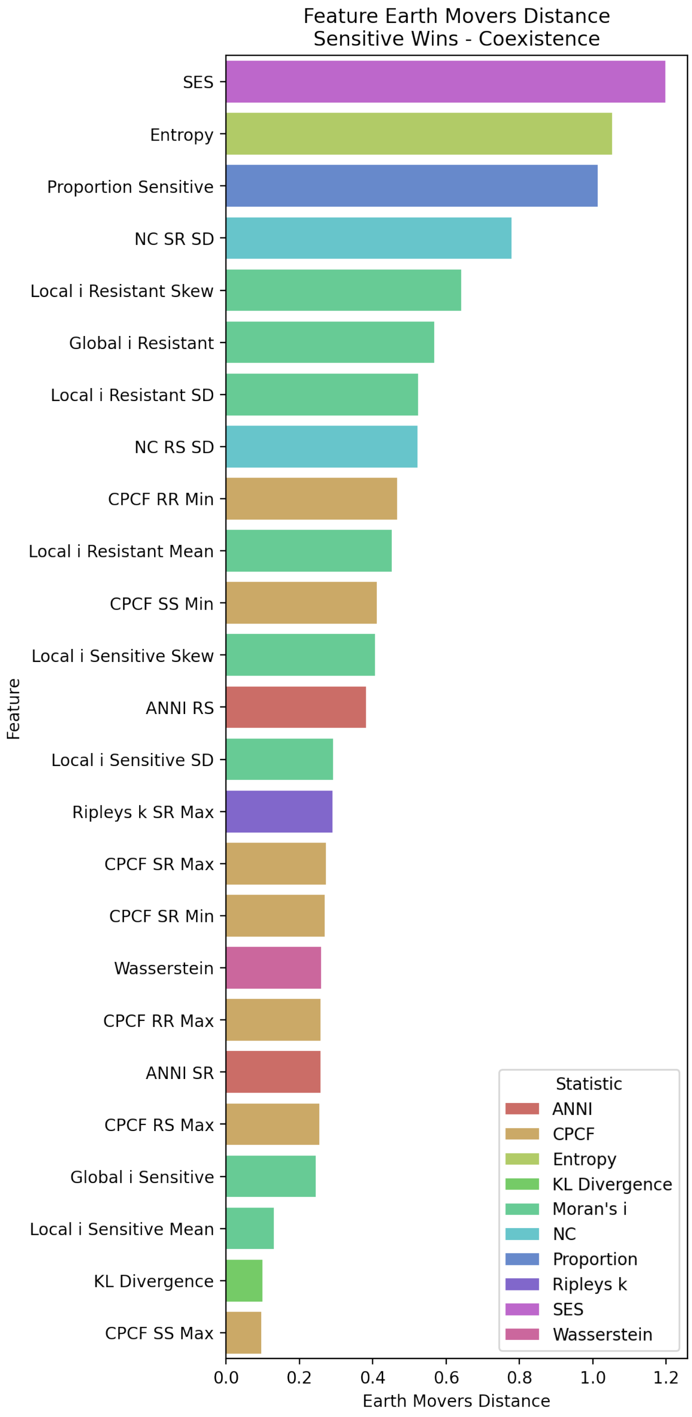

Fig 5. Full list of the distinguishability (Earth movers distance) between the feature distributions of *Sensitive Wins* and *Coexistence*.

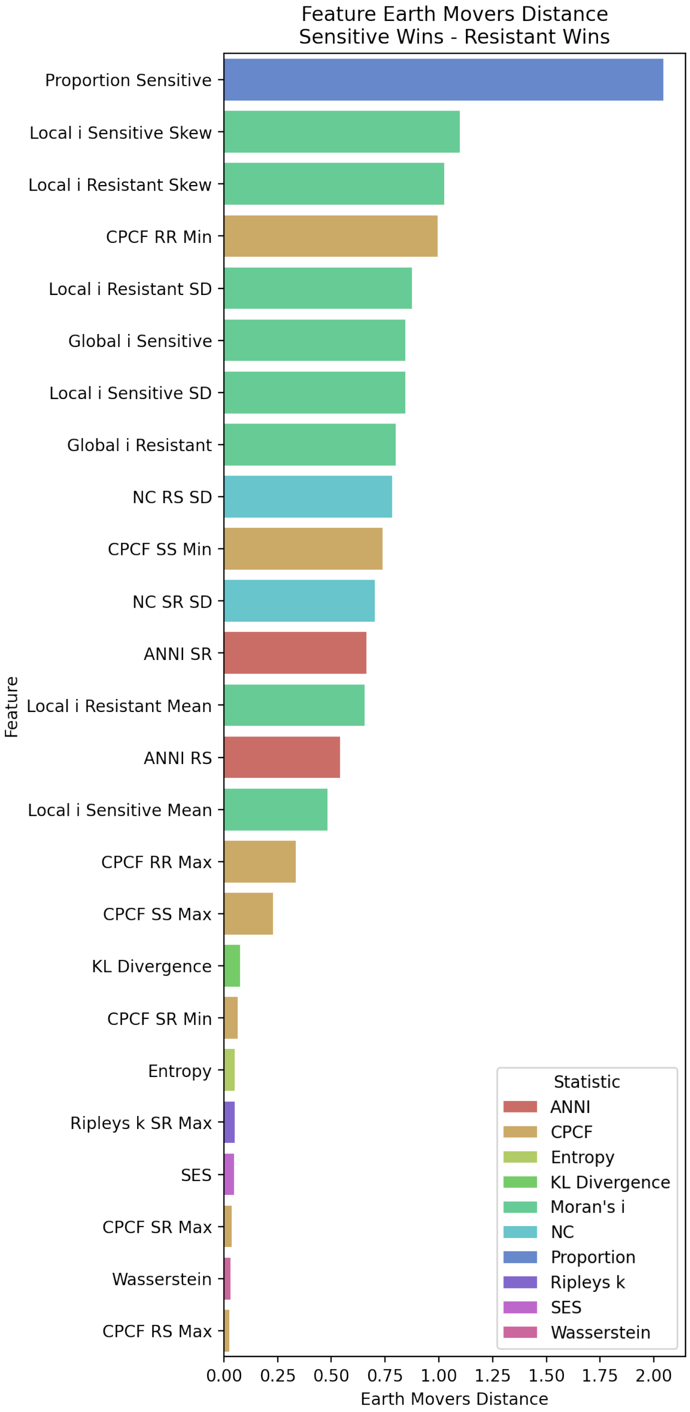

Fig 6. Full list of the distinguishability (Earth movers distance) between the feature distributions of *Sensitive Wins* and *Resistant Wins*.

4 Feature distributions

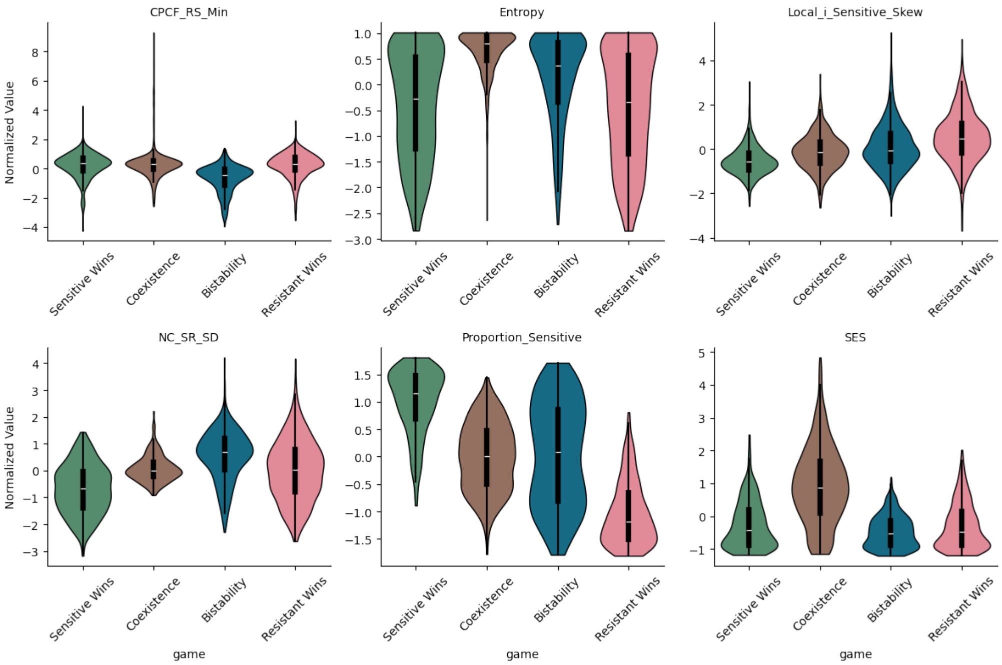

Fig 7. Feature distributions (normalized) for all features in Main Figure 3.

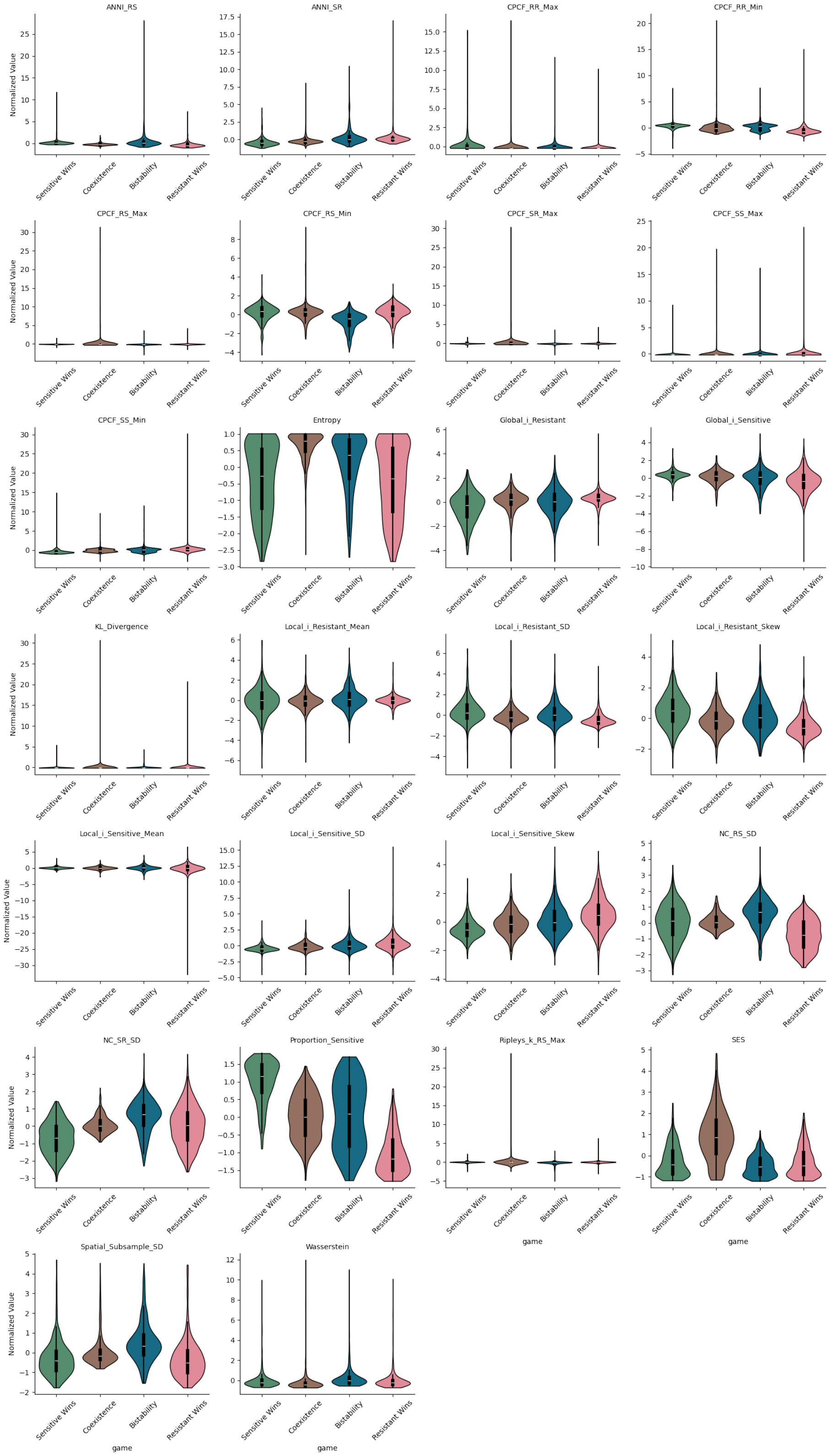

Fig 8. Feature distributions (normalized) for all non-correlated features.

5 Mutual information between feature and game

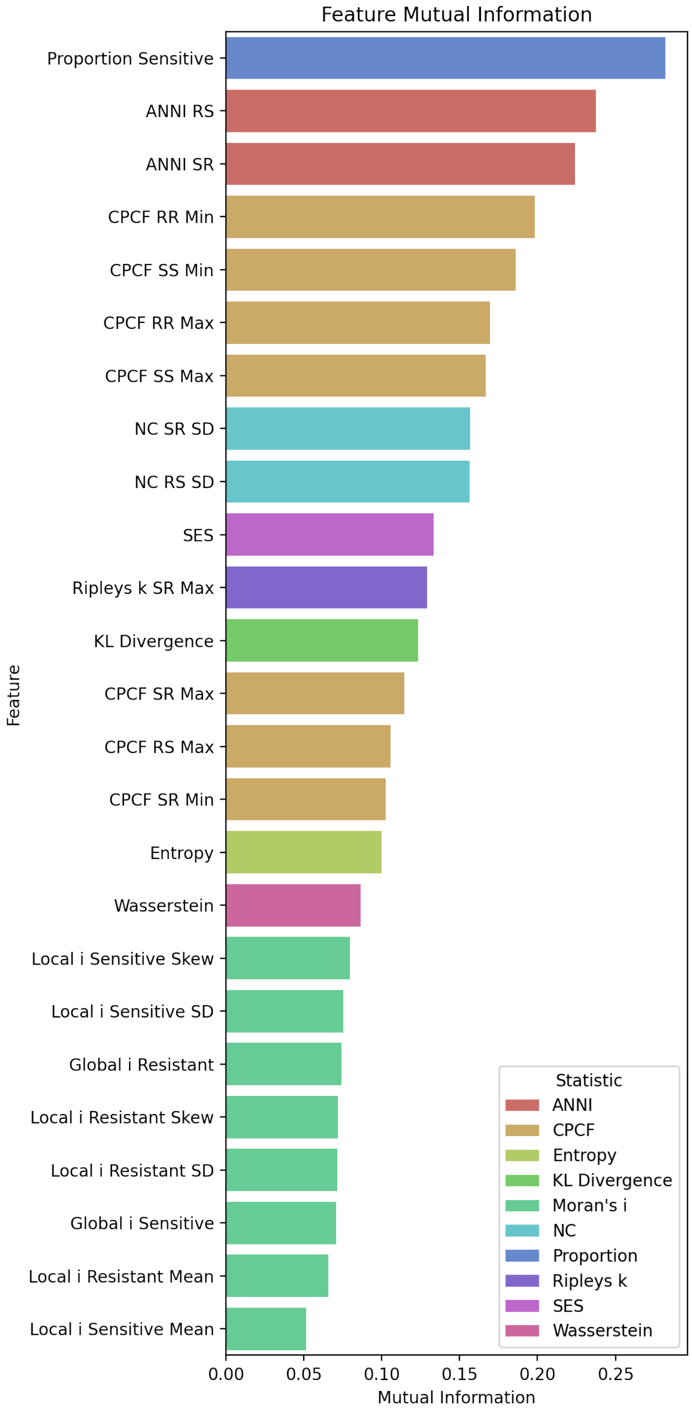

Fig 9. Mutual information between feature and game, for each feature.

6 Machine learning performance

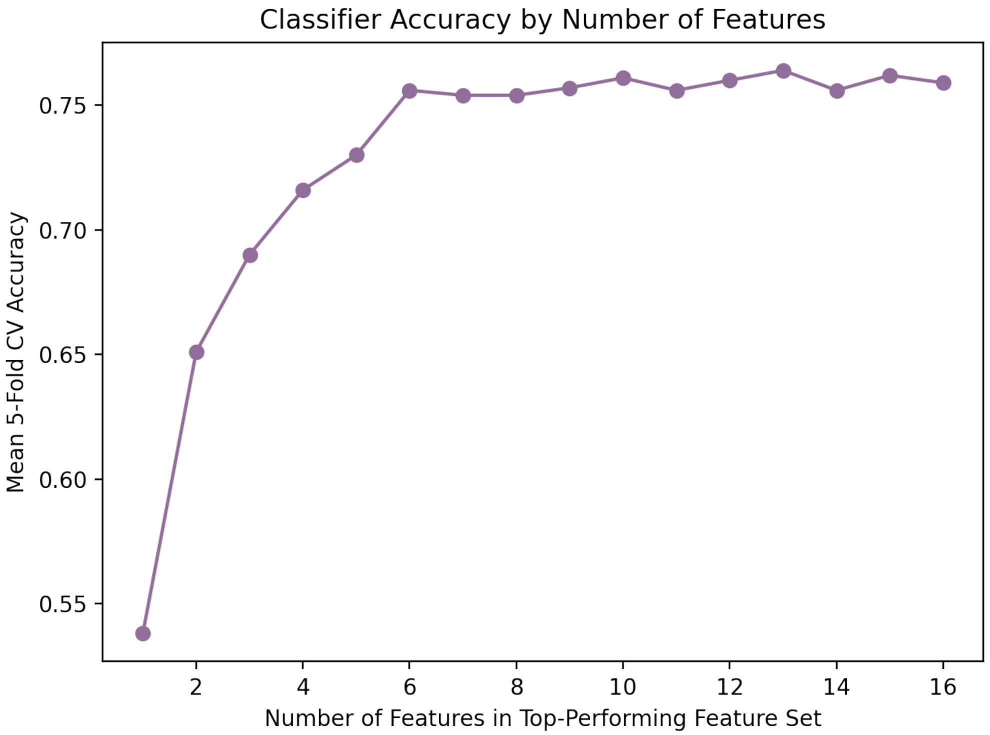

Fig 10. The 5-fold cross-validation testing accuracy of the highest performing feature set for each feature set size.

**Feature**
ANNI RS
ANNI SR
CPCF RR Max
CPCF RR Min
CPCF SR Max
CPCF SS Min
KL Divergence
Local i Resistant Mean
NC RS SD
NC SR SD
Proportion Sensitive
Ripleys k RS Max

Table 3. Top-performing feature set based on top-ten sequential feature selection. The table is ordered by the order features were included in each subsequent feature set as the features were being selected.

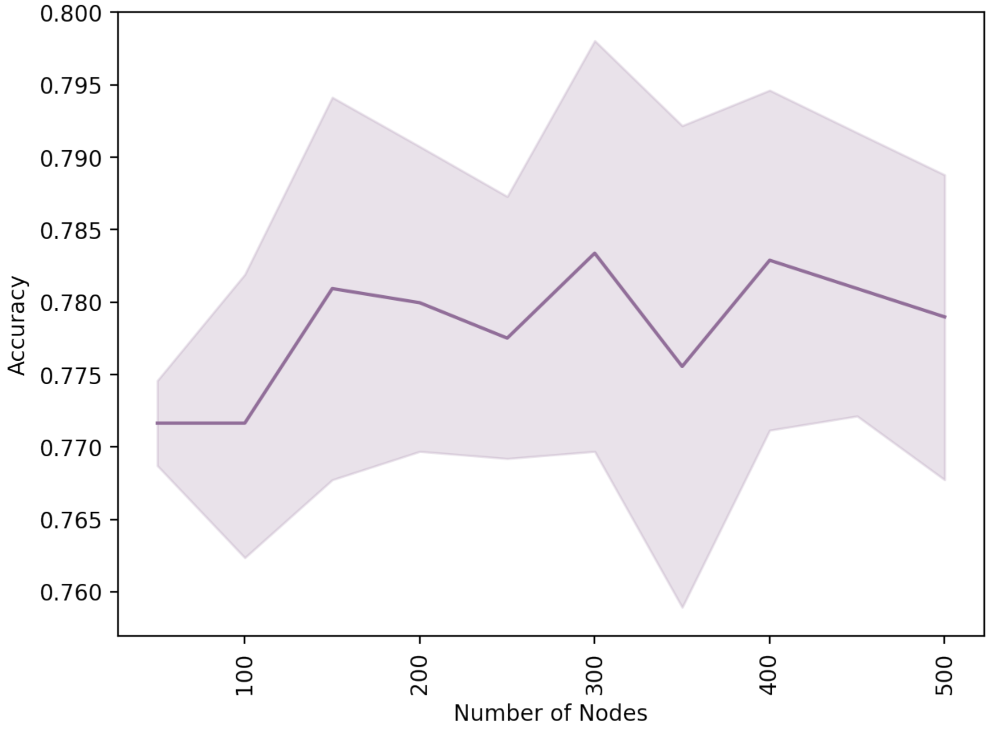

Fig 11. The 5-fold cross-validation testing accuracy of the model trained on the top 12 features across different number of nodes in the layer.

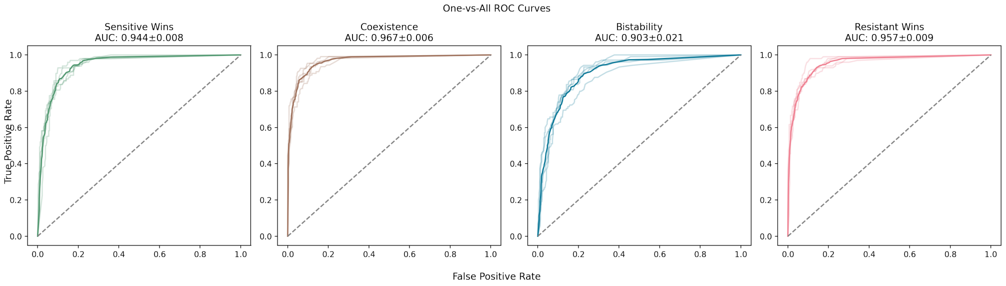

Fig 12. ROC curves from 5-fold cross-validation runs of the model.

References

1. Clark PJ, Evans FC. Distance to Nearest Neighbor as a Measure of Spatial Relationships in Populations. Ecology. 1954;35(4):445–453. doi:https://doi.org/10.2307/1931034.

2. Bull JA, Mulholland EJ, Leedham SJ, Byrne HM. Extended correlation functions for spatial analysis of multiplex imaging data. Biological Imaging. 2024;4:e2. doi:10.1017/S2633903X24000011.

3. Feng Y, Yang T, Zhu J, Li M, Doyle M, Ozcoban V, et al. Spatial analysis with SPIAT and spaSim to characterize and simulate tissue microenvironments. Nature Communications. 2023;14(1):2697. doi:10.1038/s41467-023-37822-0.

4. Shannon CE. A mathematical theory of communication. The Bell system technical journal. 1948;27(3):379–423.

5. Kullback S, Leibler RA. On Information and Sufficiency. The Annals of Mathematical Statistics. 1951;22(1):79 – 86. doi:10.1214/aoms/1177729694.

6. Scott DW. Multivariate Density Estimation: Theory, Practice, and Visualization. Wiley Series in Probability and Statistics. Wiley; 1992. Available from: https://onlinelibrary.wiley.com/doi/book/10.1002/9781118575574.

7. Moran PAP. The Interpretation of Statistical Maps. Journal of the Royal Statistical Society Series B (Methodological). 1948;10(2):243–251.

8. Stoltzfus CR, Filipek J, Gern BH, Olin BE, Leal JM, Wu Y, et al. CytoMAP: A Spatial Analysis Toolbox Reveals Features of Myeloid Cell Organization in Lymphoid Tissues. Cell Reports. 2020;31(3):107523. doi:https://doi.org/10.1016/j.celrep.2020.107523.

9. Morueta-Holme N, Blonder B, Sandel B, McGill BJ, Peet RK, Ott JE, et al. A network approach for inferring species associations from co-occurrence data. Ecography. 2016;39(12):1139–1150. doi:https://doi.org/10.1111/ecog.01892.

10. Xi J, Niles-Weed J. Distributional Convergence of the Sliced Wasserstein Process. In: Koyejo S, Mohamed S, Agarwal A, Belgrave D, Cho K, Oh A, editors. Advances in Neural Information Processing Systems. vol. 35. Curran Associates, Inc.; 2022. p. 13961–13973. Available from: https://proceedings.neurips.cc/paper_files/paper/2022/file/5a5e9197ea547141b4977a5a198bbaac-Paper-Conference.pdf.

11. Bull JA, Moore JW, Mulholland EJ, Leedham SJ, Byrne HM. MuSpAn: A Toolbox for Multiscale Spatial Analysis. bioRxiv. 2025;doi:10.1101/2024.12.06.627195.
